# Sero-antigen prevalence of lymphatic filariasis and risk factors of podoconiosis in Busiriba sub-county, Kamwenge district, Southwestern Uganda, August–September 2018

**DOI:** 10.1186/s13104-024-06801-z

**Published:** 2024-05-17

**Authors:** Vicent Mwesigye, Benson Musinguzi, Benson Okongo, William Mucunguzi, Michael Nyende Kakaire, Richard Migisha

**Affiliations:** 1 Department of Medical Laboratory Sciences Faculty of Medicine, Mbarara University of Science and Technology, P.O Box 1410, Mbarara, Uganda; 2 Department of Medical Laboratory Sciences, Faculty of Health Sciences, Muni University, P.O Box 725, Arua, Uganda; 3 District Health Office Kamwenge District, P.O Box 1408, Kamwenge, Uganda; 4 Department of Physiology, Faculty of Medicine, Mbarara University of Science and Technology, P.O Box 1410, Mbarara, Uganda

**Keywords:** Filariasis, Podoconiosis, Lymphatic filariasis, Bancroftian elephantiasis, Uganda

## Abstract

**Objective:**

Given the neglected nature of filariasis, especially in Uganda where data are scarce, this cross-sectional study aimed to determine the sero-antigen prevalence of lymphatic filariasis and risk factors associated with non-lymphatic filariasis (podoconiosis) in Busiriba Sub-county, Kamwenge District, Uganda, during August–September 2018, to inform targeted elimination efforts.

**Results:**

We enrolled 101 participants, among whom 35 (34.7%) had podoconiosis. The sero-antigen prevalence of lymphatic filariasis was 1.0%. Older age and walking barefoot were associated with increased podoconiosis risk. Specifically, individuals aged 25–49 years with had 7.38 times higher odds of podoconiosis (adjusted odds ratio [aOR] = 7.38, 95% CI: 1.36–40.13) compared to those under 25 years, while those aged ≥ 50 years had even higher odds (aOR = 8.49, 95%CI: 1.44–50.15). Additionally, individuals who reported walking barefoot had 14 times higher odds of podoconiosis (aOR = 14.08; 95% CI: 2.49–79.50).

## Introduction

Lymphatic filariasis (LF) and podoconiosis are neglected tropical diseases that impose substantial morbidity and disability on a global scale [1]. LF, attributed to the parasitic worms *Wuchereria bancrofti*, *Brugia malayi*, and *Brugia timori*, had infected an estimated 51 million individuals (43–63 million) worldwide by 2018 [2]. Podoconiosis, on the other hand, is a non-filarial form of lower limb lymphedema that occurs in individuals exposed to red clay soil derived from volcanic rock [3]. It is endemic in highland areas of tropical Africa, Central and South America, and northwest India, affecting millions of individuals and resulting in significant social and economic burdens [4]. The highest reported prevalence estimates of podoconiosis were found in Africa, with rates of 8.08% in Cameroon, 7.45% in Ethiopia, 4.52% in Uganda, 3.87% in Kenya, and 2.51% in Tanzania [5].

Despite the substantial impact of LF and podoconiosis on affected populations, they remain largely neglected in terms of research, public health intervention, and funding [4, 6]. In Uganda, data on the epidemiology of these diseases are particularly limited, hindering efforts to track progress towards elimination. In 2015, the World Health Organization (WHO) Uganda received a report of increased elephantiasis cases in Kamwenge District, later confirmed to be podoconiosis, with Busiriba Sub-county being the most affected [7]. Our study was a follow-up to these public health concerns, to further investigate the magnitude and risk factors of LF and podoconiosis, to provide evidence to guide prevention and control efforts beyond the initial concerns raised about podoconiosis in the district. This study determined the sero-antigen prevalence of LF and identified risk factors of podoconiosis in Busiriba Sub-county, Kamwenge District, southwestern Uganda. The findings of this study will contribute to filling the knowledge gap regarding these neglected tropical diseases in Uganda and inform targeted interventions for their control and elimination.

## Methods

### Study setting, study design, and study population

This cross-sectional study, was conducted in Busiriba sub-county, Kamwenge district, southwestern Uganda, approximately 400 km from Kampala. Busiriba was the most affected sub-county by elephantiasis in the district [7, 8], covering an area of approximately 319.2 square kilometers with 7 parishes (Busiriba, Kaniima, Kyakarafa, Kinoni, Bujogobe, Bigodi, and Kahondo) and 73 villages. The main socioeconomic activity in the area is mixed farming due to mineral rich volcanic soils, and the district’s general population is about 28,044 residents, comprising of various ethnic backgrounds, with Bakiga forming 46% of the population. The study population who were recruited included residents of Busiriba sub-county, Kamwenge District, who had lived in the study area for > 3 months. Study participants who were on treatment for filariasis or withdrew from the study were excluded. We excluded participants on treatment for lymphatic filariasis to avoid false negative results on Circulating Filarial Antigen Test. This study was conducted during August–September 2018. No mass drug administration (MDA) for LF had been conducted within 5 years prior to this study.

### Sample size calculation and sampling

Sample size was calculated using Kish and Leslie formula (1963) formula basing on 7.2% prevalence of filariasis in Kamwenge District study by the Programme to Eliminate Lymphatic Filariasis (PELF) in 2012 [9].

$$n=\frac{{Z}^{2} {P(1-P)}}{d^{2}}$$

n = study sample size required.

Z = critical value associated with 95% confidence interval = 1.96.

P = estimated proportion of an attribute that is present in the population, (0.072)

d = margin of error = 0.05.

Substituting in the above formula *n* = 103 participants.

A stratified sampling procedure was used, with Busiriba Sub County stratified into seven parishes. Homes in each parish were listed and participants were randomly selected. Blood samples were collected from randomly selected participants who consented to participate in the study, and a structured questionnaire was used to collect data on socio-demographic, and behavioral data. For LF, we collected data on potential risk factors such as sleeping under treated mosquito nets, closing doors and windows early, and living near forests or swamps. For podoconiosis, the risk factors included barefoot exposure to soils.

### Laboratory methods

Blood samples were collected from participants using conventional venipuncture technique and tested for Circulating Filarial Antigen (CFA) using Immunochromatographic test (ICT) (Alere Scarborough, Inc. 10 Southgate Road, Scarborough, ME 04074 USA).

Soil samples were also taken and tested for the presence of silicon compounds using Inductively Coupled Plasma (ICP) at the College of Agriculture and Animal Husbandry Laboratory, Makerere University. Briefly, soil samples were air-dried and then sieved into 75 mL plastic extraction bottles, with 10 cm3 of soil in each. 25 mL of 0.5 M acetic acid extracting solution was added to each bottle and left overnight. The mixtures were then shaken for 50 min, filtered, and the extract collected. Silicon concentrations in the soil extracts were measured using ICP optical emission spectrometry after standardization.

### Case definition

We defined a suspected case of podoconiosis as the onset of hard swelling of the lower limbs lasting for at least one month prior to the study period, in a resident of Busiriba Sub-county, accompanied by any of the following associated symptoms: lower limb skin itching, burning sensations, plantar edema, hyperkeratosis, rigid toes, formation of mossy papillomata, or skin nodules on the lower limbs [7]. A confirmed case was a suspected case with a negative result on the immunochromatographic card test (ICT) for microfilaria antigen; a negative ICT result rules out LF, thereby confirming the diagnosis of podoconiosis [10, 11]. All suspected case persons underwent clinical examination by a medical doctor to rule out other potential diagnoses, including leprosy and other dermatological disorders.

### Data analysis

Data was entered into EpiData 3.1 software (EpiData, Odense, Denmark) and then exported to STATA version 13 (StataCorp, College Station, Texas, USA) for analysis. Bivariate analysis was performed to evaluate associations between independent and dependent variables, and multivariate logistic regression models were used to obtain the final predictive model of adjusted covariates significantly associated with podoconiosis. All variables with *p* < 0.2 at bivariate analysis were included in the multivariable model. Variables in the final model with *p* < 0.05 were considered statistically significant.

## Results

### Characteristics of study participants

The study enrolled 101 participants; we excluded 2 individuals who did not consent. Most (61.4%) were female. The mean age of the participants was 41 (± 20) years, with most (*n* = 44; 43.6%) aged between 25 and 49 years. The majority (62.4%) had lived in the area for ≥ 30 years. The majority (74.3%) of the participants walked barefooted (Table 1). A higher proportion of participants with podoconiosis were in the age group < 25 years (8.6% vs. 28.8%; *p* = 0.06) compared to those without the condition. The duration of residence also differed significantly, with a higher proportion of individuals with podoconiosis residing in the area for ≥ 30 years (77.1% vs. 54.6%; *p* = 0.026). Additionally, a significantly higher proportion of individuals with podoconiosis reported walking barefooted (94.3% vs. 63.6%; *p* = 0.001) compared to those without the condition (Table 1).

**Table 1:** Socio-demographic and behavioral characteristics of study participants

Characteristic	Overall (*N* = 101)	Had podoconiosis (*n* = 35)	No podoconiosis (*n* = 66)	*p* value
	*n* (%)	*n* (%)	*n* (%)	
Age category in years*				0.06
< 25	22 (21.8)	3 (8.6)	19 (28.8)	
25–49	44 (43.6)	17 (48.6)	27 (40.9)	
≥ 50	35 (34.7)	15 (42.9)	20 (30.3)	
**Sex**				0.286
Male	39 (38.6)	16 (45.7)	23 (34.9)	
Female	62 (61.4)	19 (54.3)	43 (65.1)	
**Education level**				0.726
None	36 (35.6)	14 (40.0)	22 (33.3)	
Primary	57 (56.4)	18 (51.4)	39 (59.1)	
Secondary	8 (7.9)	3 (8.6)	5 (7.6)	
**Duration of residence in years**				0.026
< 30	38 (37.6)	8 (22.9)	30 (45.5)	
≥30	63 (62.4)	27 (77.1)	36 (54.6)	
**Ever heard about the disease**				0.463
No	30 (29.7	12 (34.3)	18 (27.3)	
Yes	71 (70.3)	23 (65.7)	48 (72.7)	
**Ever got health education on the disease**				0.153
No	75 (74.3)	23 (65.7)	52 (78.8)	
Yes	26 (25.7)	12 (34.3)	14 (21.2)	
**Walking barefooted**				0.001
No	26 (25.7)	4 (4.6)	24 (36.4)	
Yes	75 (74.3)	33 (94.3)	42 (63.6)	
**Sleeping under treated mosquito net**				0.454
No	12 (11.9)	3 (8.6)	9 (13.6)	
Yes	89 (88.1)	32 (91.4)	57 (86.4)	
**Close doors/windows early**				0.405
No	16 (15.8)	7 (20.0)	9 (13.6)	
Yes	85 (84.2)	28 (80.0)	57 (86.4)	
**Live near forest/swamp**				0.594
No	40 (39.6)	12 (34.3)	28 (42.4)	
Yes	61 (60.4)	23 (65.7)	38 (57.6)	

*Mean age = 41 (± 20) years; **Median duration = 24 (interquartile range [IQR]: 14–34) years

### Sero-antigen prevalence of lymphatic filariasis

Among 101 participants surveyed, one (1%) tested positive for filarial antigen, for a sero-antigen prevalence of 1%. The individual who tested positive was a female participant with lymphedema.

### Risk factors for podoconiosis

In the multivariate analysis (Table 2), there was a notable increase in the odds of podoconiosis with increasing age. Participants aged 25–49 years had higher odds compared to those under 25 years (adjusted odds ratio [aOR] 7.38, 95% CI: 1.36–40.13, *p* = 0.021), and those aged 50 years and above showed even higher odds (aOR 8.49, 95% CI: 1.44–50.15, *p* = 0.018). Additionally, individuals who reported walking barefoot had significantly higher odds of podoconiosis compared to those who did not (aOR 14.08, 95% CI: 2.49–79.50, *p* = 0.003).

**Table 2:** Risk factors for podoconiasis, Busiriba Sub-county, Kamwenge District, Uganda, August – September 2018

Characteristic	% podoconiosis positive		Bivariate analysis			Multivariate analysis	*P* value
	*n*/*N* (%)		OR (95%CI)		*P* value	Adjusted OR (95%CI)	
**Age category (years)**							
<25 yrs	3/22 (13.6)		Ref			Ref	
25–49 yrs	17/44 (38.6)		3.99 (1.02–15.54)		0.046	7.38 (1.36–40.13)	0.021
50 yrs & above	15/35 (42.86)		4.75 (1.48–19.06)		0.028	8.49 (1.44–50.15)	0.018
**Sex**							
Male	16/39 (41.0)		Ref			Ref	
Female	19/62 (30.7)		0.64 (0.28–1.47)		0.287	0.36 (0.12–1.07)	0.065
**Education level**							
None	14/36 (38.9)		Ref				
Primary	18/57 (31.6)		0.73 (0.30–1.74)		0.47		
Secondary	3/8 (37.5)		0.94 (0.19–4.58)		0.942		
**Duration of residence in years**							
<30		8/38 (21.1)	Ref				
≥30		27/63 (42.9)	2.81 (1.11–7.10)		0.029		
**Walking barefooted**							
No		2/26 (7.7)	Ref			Ref	
Yes		33/75 (44.0)	9.43 (2.08–42.80)		0.004	14.08 (2.49–79.50)	0.003
**Ever got health education on the disease**							
No		23/75 (30.7)		Ref		Ref	
Yes		12/26 (46.2)		1.94 (0.78–4.83)	0.156	1.37 (0.51–3.70)	0.533
**Live near swamp/forest**							
No		12/40 (30.0)		Ref			
Yes		23/61 (37.7)		1.41 (0.60–3.31)	0.427		
**Close doors/windows early**		7/16 (43.8)		Ref			
No		28/85 (32.9)		0.63 (0.21–1.87)	0.407		
Yes							

Ref: Reference category; OR: Odds Ratio; CI: Confidence interval

### Soil analysis for silicon concentration

The mean free silicon concentration in the 40 samples tested was 36.1 ppm (mg/kg of soil). The average pH of the samples was 6.2, with a range of 5.6 to 6.8.

## Discussion

This study in Busiriba Sub-county, Kamwenge District, Uganda, determined the sero-antigen prevalence of LF and risk factors of podoconiosis. LF was found to have a low prevalence of 1.0% in the area. Walking barefoot and older age (≥ 25 years) were identified as risk factors for podoconiosis. Soil testing revealed that the soils contained silicon and were acidic. Overall, the findings point towards the co-existence of both filarial and non-filarial forms of elephantiasis in the study area. However, there has been significant progress towards the elimination of LF.

The prevalence of filarial elephantiasis in the current study was found to be 1%, consistent with a similar study in Kenya that reported a prevalence of 1.3% [12]. However, the 1% prevalence in Busiriba Sub-county is significantly lower than that observed in Northern Uganda, where circulating filarial antigen positivity ranged from 18 to 30% in different communities [13]. This discrepancy is linked to ongoing interventions such as Mass Drug Administration (MDA) with ivermectin and albendazole and the distribution of insecticide-treated mosquito nets aimed at controlling the disease in endemic regions [14]. As such, the low prevalence of non-filarial elephantiasis, is an indicator of progress towards its elimination.

Walking barefoot was identified as a risk factor for podoconiosis. This is consistent with findings from studies in other sub-Saharan Africana countries including Ethiopia and Kenya [15–19]. Walking barefoot is known to predispose to irritant volcanic red clay soils [6]. Moreover, high silicon levels were identified in soil samples in the current study. Soil rich in silicon, along with elevated levels of incompatible elements and high concentrations of phyllosilicate clays, mica groups, quartz (crystalline silica), iron oxide, and zirconium, can penetrate the skin, triggering inflammation and the development of lymphedema [4, 20].

In our study, older participants showed higher odds of podoconiosis compared to those under 25 years old. This aligns with findings from studies in sub-Saharan Africa [15, 18, 19, 21]. This trend may be attributed to prolonged exposure to irritant soils as individuals age. Disease development is a gradual process, leading to a higher accumulation of cases in older age groups.

### Limitations

The limitations of the study include the lack of blood smear examination, which is the gold standard for diagnosing filarial worms. We did not do blood smear examinations due to the periodicity of the worms and the timing of blood sample collection during the day. However, this is unlikely to significantly affect the reported prevalence. Additionally, the antigen used in the ICT test originated from India, and genetic variation within and between lymphatic filariasis-causing agents could have influenced the test’s sensitivity. Furthermore, the study may be prone to information bias from participants, particularly social desirability bias, as much of the exposure data were self-reported. This potential bias could have led to associations being biased towards the null. Finally, we acknowledge that we may have been underpowered to comprehensively investigate the risk factors of podoconiosis, as we did not conduct a specific sample size calculation for this study objective. Accordingly, the analysis of risk factors of podoconiosis was exploratory in nature due to the lack of pre-planned power calculation.

## Conclusion

In conclusion, our study identified the coexistence of non-filarial (Podoconiosis) and filarial elephantiasis in Busiriba Sub-county. The low prevalence of filarial elephantiasis (1%) suggests significant progress towards its elimination in the region. Walking barefoot, emerged as a significant risk factor for podoconiosis, with older age groups having higher prevalence of podoconiosis. These findings underscore the importance of wearing shoes, especially from an early age, to reduce the incidence of podoconiosis in this area.

## Data Availability

The datasets used and/or analyzed during the current study is available from the corresponding author on reasonable request.

## Abbreviations

aOR: Adjusted odds ratio
CFA: Circulating Filarial Antigen
ICT: Immunochromatographic test
IQR: Interquartile range
LF: Lymphatic filariasis
MDA: Mass drug administration
OR: Odds ratio
PELF: Programme to Eliminate Lymphatic Filariasis
WHO: World Health Organization
SD: Standard deviation
